# Development of a mobile APP to promote physical activity in individuals with knee osteoarthritis: the “Move for Knee”™ study protocol

**DOI:** 10.3389/fresc.2025.1540320

**Published:** 2025-05-30

**Authors:** Sara Liguori, Viviana Andreozzi, Antimo Moretti, Roberta Angari, Marco Paoletta, Giovanni Iolascon, Francesca Gimigliano

**Affiliations:** ^1^ Department of Medical and Surgical Specialties and Dentistry, University of Campania “Luigi Vanvitelli”, Naples, Italy; ^2^ Department of Mental and Physical Health and Preventive Medicine, University of Campania “Luigi Vanvitelli”, Naples, Italy; ^3^ Department of Architecture and Industrial Design, University of Campania “Luigi Vanvitelli”, Caserta, Italy

**Keywords:** knee, osteoarthritis, digital health, physical activity, pain

## Abstract

In modern society, physical activity is essential for maintaining both physical and mental health, particularly as technological advancements have contributed to a sedentary lifestyle. The World Health Organization recommends a minimum of 150 minutes of moderate exercise weekly for adults; however, many individuals, especially those with knee osteoarthritis (KOA), do not meet these guidelines. KOA, the most common form of arthritis, affects millions globally and leads to significant disability, pain, and reduced quality of life. The condition is exacerbated by sedentary behavior and high body mass index (BMI). Educational interventions promoting lifestyle changes, including increased physical activity, have shown effectiveness in managing KOA. To address the low adherence rates to physical activity guidelines among KOA patients, the “Move for Knee”™ mobile app was developed. This app offers personalized exercise programs based on user-input data about lifestyle and activity levels, aiming to enhance patient engagement and adherence. Its features include exercise tracking, real-time monitoring, and communication with healthcare providers through an integrated chat function. A randomized controlled trial (RCT) will evaluate the app's efficacy in improving pain, function, and quality of life among individuals aged 45–70 with KOA. The study will compare outcomes from users of the app against a control group receiving standard advice on physical activity. Preliminary expectations suggest that the app will improve adherence to exercise recommendations and overall management of KOA, while also providing cost-effective healthcare solutions. The ultimate goal is to empower patients, enhance their self-management capabilities, and improve their overall health outcomes through an innovative, technology-driven approach. Future research will focus on assessing the app's clinical effectiveness and usability across various patient demographics.

## 1 Introduction

Physical activity, in modern society, is key to ensure physical and mental well-being, preventing disabling chronic conditions, and improving overall health and quality of life (1).

The evolution of society, particularly advancements in technology, has progressively reduced the amount of physical activity and daily energy expenditure, with significant negative effects on global health (2).

The World Health Organization (WHO) recommends at least 150 minutes of moderate physical activity per week for adults. However, many individuals fail to meet even this minimum threshold. Lack of exercise is considered a significant risk factor for many conditions, including obesity, type 2 diabetes, cardiovascular diseases, hypertension, and degenerative joint disorders, particularly knee osteoarthritis (3).

Knee osteoarthritis (KOA) is the most prevalent type of arthritis and a highly disabling condition (4). In 2023, approximately 364.58 million prevalent cases of KOA were recorded worldwide, with 29.51 million incident cases, and 11.53 million disability-adjusted life years (5, 6) attributed to the disease. Patients with KOA commonly experience knee pain, limited morning stiffness, reduced functionality, progressive decline of activities of daily living (ADL), decreased quality of life, and significant healthcare resource utilization (7, 8). Both constitutional and mechanical factors contribute to the onset and progression of this degenerative joint disease, such as high body mass index (BMI) or sedentary behavior. Among the recommended interventions for KOA, educational and lifestyle advice, including weight management and physical activity, represent an effective therapeutic strategy to improve clinical condition and functional outcomes (9, 10). Despite these recommendations, less than 20% of men and fewer than 10% of women with KOA adhere to the prescribed physical activity guidelines (11).

To improve adherence to physical activity in people with KOA, a personalized approach has been suggested. This approach involves customizing physical activity goals, monitoring progress in real-time, creating a sustainable environment, and fostering a supportive social network to promote an active lifestyle (12). These measures could encourage patients to engage in higher levels of physical activity. In this context, digital health technologies have proven to be as effective as traditional treatments in patient education, physical activity promotion, and exercise interventions while also being cost-effective for the KOA population.

The aim of this study is to develop and implement a mobile app, “Move for Knee”™, featuring specific physical activity programs designed for Italian individuals with KOA. Furthermore, this paper describes the study protocol of the RCT designed to investigate the efficacy of the clinical application of app for managing KOA.

## 2 Materials and methods

### 2.1 The “Move your Knee”™ app

The mobile APP called “Move Your Knee”™ is divided into four main sections:

1) Know Your Knee: this section collects patient-entered data to help understand their lifestyle habits, daily physical activity levels, and information related to KOA. The data related to the symptoms experienced by patients and their intensity are reported through a questionnaire based on selected categories from the International Classification of Functioning, Disability, and Health (ICF) brief core set related to osteoarthritis (OA) (Table 1). Finally, the individual's level of physical activity is self-assessed using the International Physical Activity Questionnaire (IPAQ).

**Table 1:** Categories belong to the brief ICF core Set for osteoarthritis-OA (in red those specific to lower limb OA).

Categories of the component “body functions”:
ICF code ICF category title
2nd level
b280 sensation of pain
b710 mobility of joint functions
b730 muscle power functions
Categories of the component “body structures”:
ICF code ICF category title
s730 structure of upper extremity
s750 structure of lower extremity
s770 additional musculoskeletal structures related to movement
Categories of the component “activities and participation”:
ICF code ICF category title
d445 hand and arm use
d450 walking
d540 dressing

At the end of the questionnaire, the app's integrated algorithm calculates the patient's estimated level of physical activity. Based on this, the app assigns a specific exercise program, including detailed execution parameters (e.g., weekly frequency) and advice for safely performing the exercises to achieve specific functional goals.

The exercises available are selected according to national and international recommendations and guidelines for KOA (13, 14). As suggested by the app, it is important for patients to consider their symptom severity before starting the exercise.

2) Move Your Knee: in this section, patients can select the difficulty/intensity level for their exercises. Advanced levels are locked until earlier objectives are achieved.

The app offers five levels, each represented by an evocative image reflecting the functional progression and user type: *Hawk, Tiger, Bull, Turtle, Sloth*.

Once an exercise program is initiated, the app provides detailed instructions for each exercise, including duration, sets and repetitions. Using a motion capture function, patients uses device's camera to record their exercises to ensure proper and safe execution. This feature enables users to perform exercises and share their execution with the doctor to monitor whether they are being done correctly and safely. The captured data is securely stored within the app and can be shared with healthcare providers through the chat feature, allowing them to provide tailored feedback and adjust exercise programs as needed.

3) Step by step: this section allows patients to track and monitor their daily progress, providing a real-time overview of their achievements.
4) Chat: to facilitate direct communication with the doctor, the app includes a native instant messaging service. Patients can contact their doctor by communicating, share files, and upload videos for better monitoring and feedback.

The system is complemented by a control panel designed for doctors, accessible via a terminal or PC. The control panel provides the following functionalities:

1) patient registration on a dedicated web platform;
2) access to patient files linked to the referring doctor;
3) monitoring of patient progress to ensure adherence to the program;
4) access to chat feature for direct communication.

**Figure 1:**
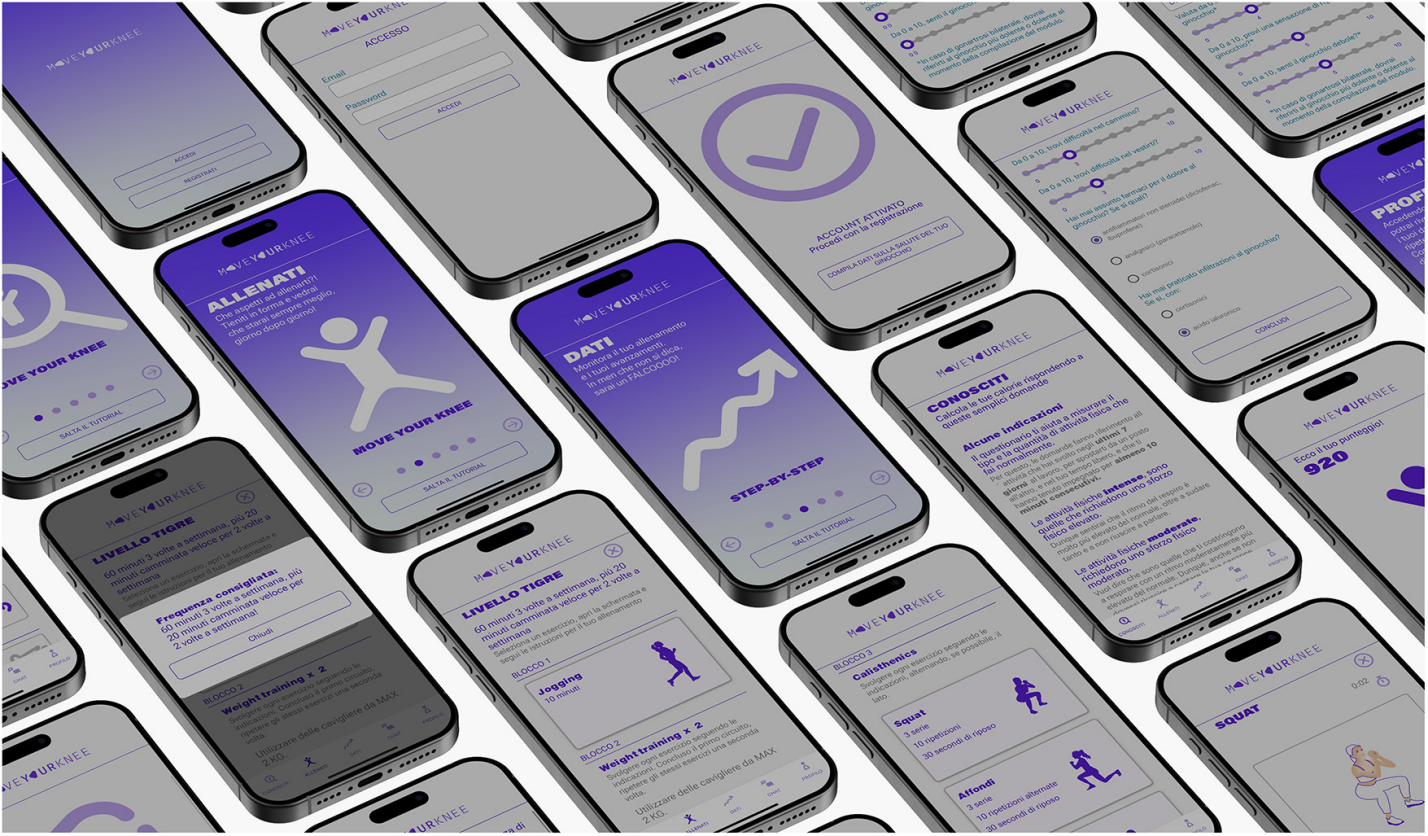
Visual overview of the “move for knee”™ app.

**Figure 2:**
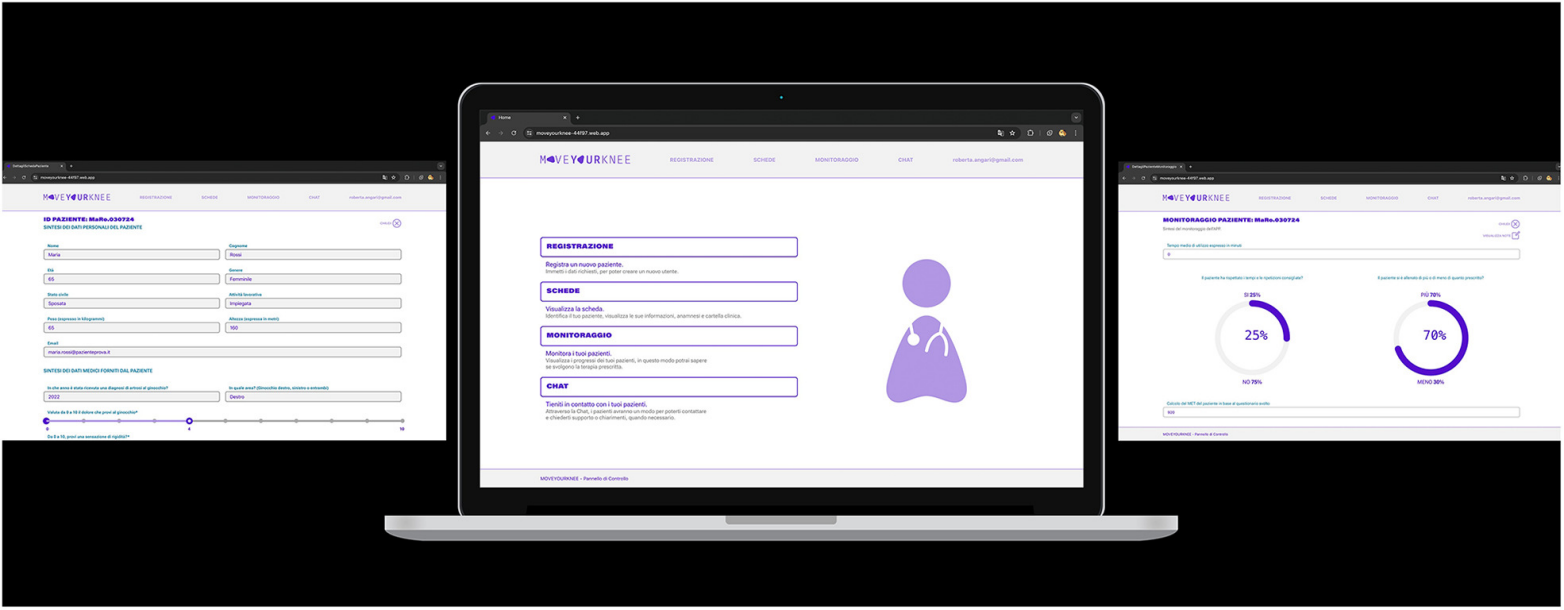
Visual overview of the medical website page linked to the app.

**Figure 3:**
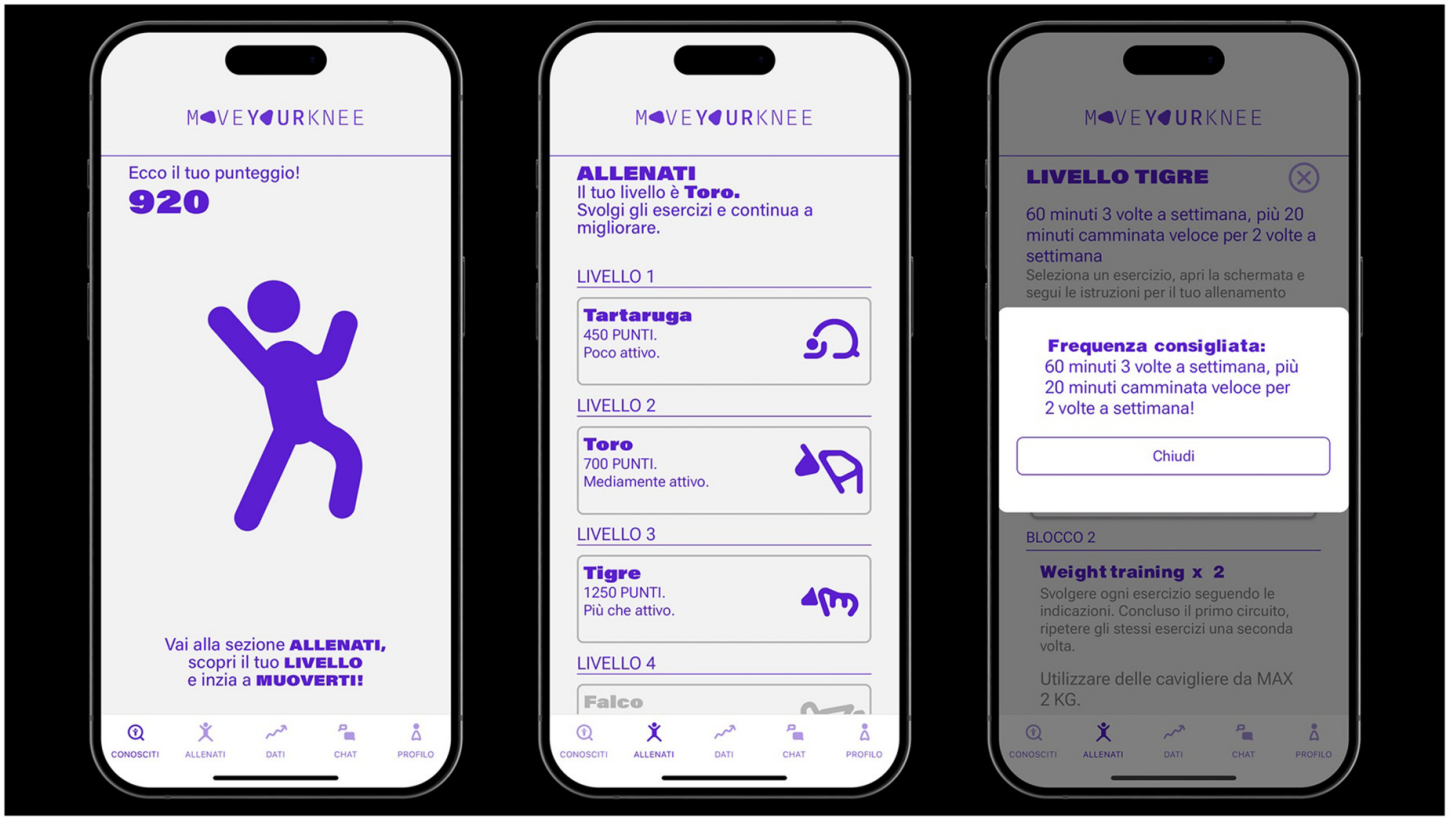
Levels of physical activity proposed.

**Figure 4:**
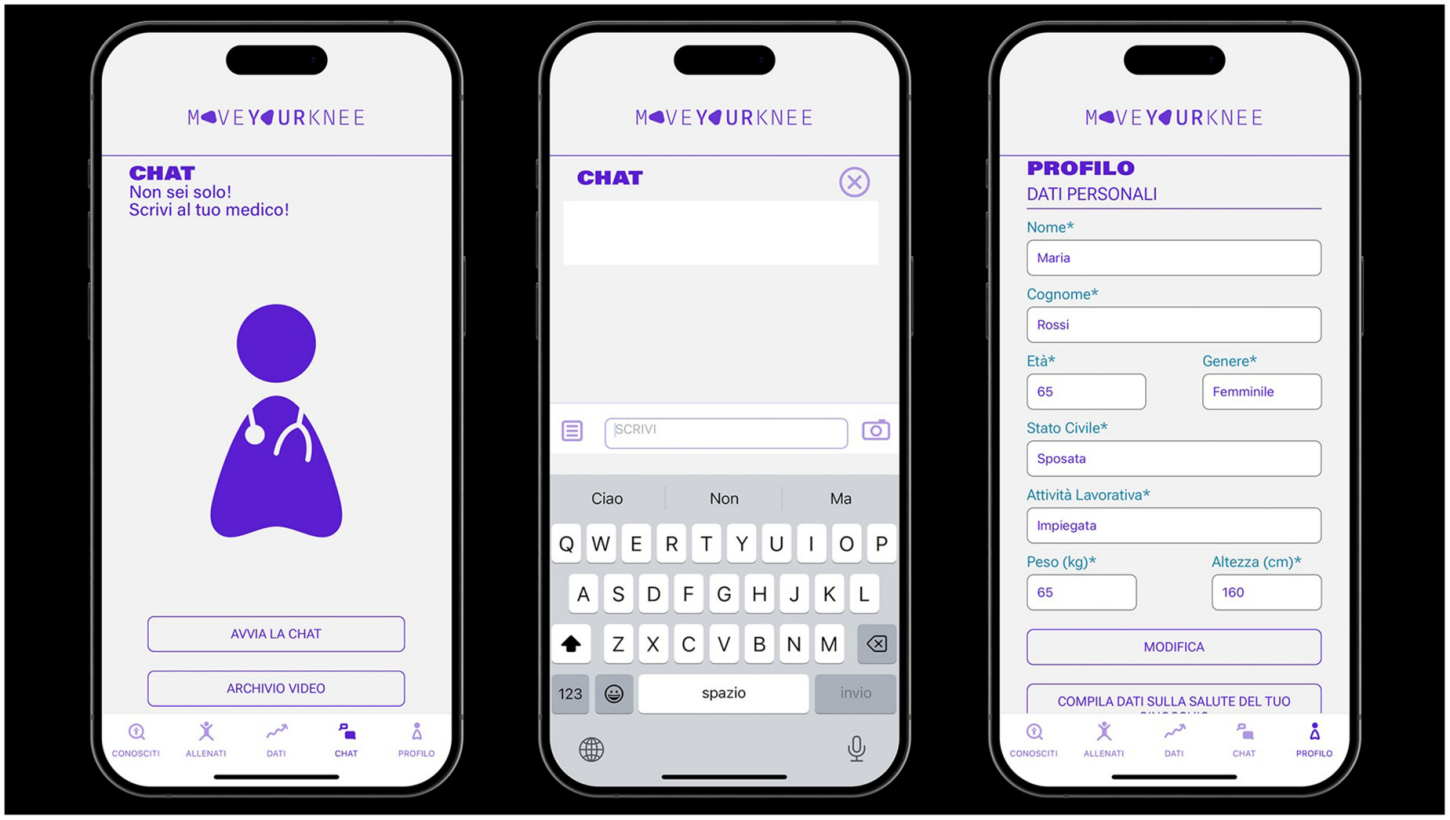
Details of the medical chat feature within the “move for knee”™ app.

Figures 1–4 provide a detailed visual description of the app and the control panel.

### 2.2 Study protocol description

#### 2.2.1 Participants

This no-profit, interventional, double-arm, monocentric study, will include men and women aged 45–70 years with unilateral or bilateral tibiofemoral KOA, diagnosed according to the American College of Rheumatology criteria for KOA, and radiographic findings (Kellgren-Lawrence grade 1–3).

Potential participants will be referred to the Physical Medicine and Rehabilitation Unit at the University of Campania “Luigi Vanvitelli.” Referrals may come from general practitioners, orthopedic specialists, or rheumatologists who identify eligible candidates based on the inclusion criteria. All participants will be required to provide informed consent before enrollment in the study, ensuring they understand the study's purpose, procedures, potential risks, and benefits. The study will adhere to good scientific and clinical practices as outlined in the Declaration of Helsinki. The Ethical Committee of the University of Campania “Luigi Vanvitelli” (Committee's reference number: Prot. 0008758/i-21/03/2024) has approved the study. The study protocol has been registered in ClinicalTrial.gov (NCT06721208).

Inclusion and exclusion criteria are reported in Table 2.

**Table 2:** Inclusion and exclusion criteria.

Inclusion criteria
• Patients with unilateral or bilateral tibiofemoral osteoarthritis according to the American College of Rheumatology classification criteria for osteoarthritis, aged between 45 and 70 years, with radiographic findings (Kellgren-Lawrence grade 1–3)
• Expression of consent to participate in the study through a signed informed consent form
• Ability to understand and use the APP through a practical demonstration in an outpatient setting
Exclusion criteria
• Psychiatric disorders that could potentially invalidate informed consent
• Pregnancy or breastfeeding

#### 2.2.2 Intervention

The study is designed as a double-arm interventional trial, indicating that participants will be divided into two groups for comparison:

- patients who will use the “Move for Knee” APP (intervention arm);
- patients who will be advised to engage in unsupervised physical activity (control arm).

Participants who meet the inclusion criteria and provide informed consent will be randomly assigned to one of the two intervention groups. The randomization process will be performed using a consecutive 1:1 allocation ratio (intervention:control) and it may involve the use of computer-generated random numbers or a randomization table to ensure unbiased assignment.

#### 2.2.3 Outcome

The following primary outcome measures will be conducted:

• Multidimensional pain evaluation to evaluate qualitative and quantitative aspects of pain:
  ○ Brief Pain Inventory (BPI), including severity and interference indices (BPI-SI, BPI-II).
  ○ Leeds Assessment of Neuropathic Symptoms and Signs (LANSS) scale for the neuropathic pain component.
• Stiffness and function to measure improvements in mobility and daily activities:
  ○ Western Ontario and McMaster Universities Osteoarthritis Index (WOMAC).

As secondary outocome measure it will be performed:

• Quality of life assessment through:
  ○ EUROQoL-5 Dimension (EQ-5D), to evaluate overall well-being and health status

Outcome measures will be collected at baseline and at specified intervals throughout the study, such as at 30 (T1), 90 (T2), and 180 days (T3) post-enrollment, to assess both short-term and long-term effects of the interventions.

For patients in the intervention group, user experience and adverse effects of the app will be evaluated using an open-ended question: “*Describe your experience with the app.*” This feedback will be collected at T1, T2, and T3.

## 3 Results

The development of the “Move Your Knee”™ mobile app aims to improve the management of KOA by providing an innovative approach to engage patients in self-management and encourage adherence to recommended physical activity. This project seeks to help individuals affected by KOA to adopt a healthier lifestyle by increasing their physical activity habits, through an easy-to-use mobile app. The “Know your Knee” section of the app collects patient data, empowering individuals with KOA to take an active role in their healthcare decisions, via a patient-centered approach. Since people with KOA often lead sedentary lifestyles and may lack adequate information about the appropriate levels of physical activity needed to safely improve their mobility and independence, the expected outcomes of this initiative are designed to address these gaps. The experimental study is expected to demonstrate high levels of adherence to and usability of the app, largely due to the training provided by physical therapists. The app will also deliver weekly feedback on physical activity performed, enhancing user engagement. To encourage compliance, users will be associated with a visual representation that corresponds to their activity level (e.g., “Hawk,” “Tiger,” “Bull,” “Turtle,” “Sloth”). Additionally, the app includes a chat function that provides a direct link to healthcare professionals, functioning as an “e-referral” system and enhancing user experience and satisfaction. Ultimately, the routine use of this app is expected to produce measurable health benefits for patients, offering a favorable cost-to-benefit ratio compared to alternative treatments.

## 4 Discussion

Physical activity is a cornerstone of a healthy lifestyle and an essential intervention for preventing and managing various health conditions. Regular exercise supports weight control, enhances cardiorespiratory fitness, and reduces the risk of disabling chronic diseases such as heart disease, diabetes, certain cancers, and degenerative musculoskeletal conditions, including KOA. Incorporating physical activity into daily routines improves its effectiveness and compliance (2).

The development of digital health technologies offers a great opportunity to engage people in physical exercise and promote healthier lifestyle habits. These tools can be particularly beneficials for individuals with KOA, helping them better manage their condition. Currently, pedometers are among the simplest and most widely used tools for measuring physical activity levels in different population. However, they lack the capacity to deliver personalized physical activity programs (15).

An ideal approach not only quantifies the physical activity performed, but also proposed a tailored program to enhance the levels and types of activity. Moreover, direct communication with healthcare professionals provides psychological support, boosting adherence to the program (16).

The proposed educational approach offers evidence-based, standardized profiles and personalized physical activity goals aligned with a patient-centered healthcare delivery model. This strategy benefits both patients and healthcare professionals, facilitating self-management of the disease and fostering better communication. Additionally, it helps increase patients’ awareness of modifiable risk factors for KOA, while identifying signs of non-adherence to recommended lifestyle changes.

A recent systematic review on the effectiveness of mobile applications for enhancing therapeutic exercise in managing knee osteoarthritis (KOA) in adults aged 40 and above concluded that these applications could be a promising supplementary option for KOA management, particularly for individuals with limited access to traditional care. The reviewed mHealth applications included features such as dietary tracking, physical activity monitoring, motivational feedback, exercise routines, and personalized advice (17). In this context, the “Move for Knee”™ app appears innovative, incorporating intuitive and user-friendly features to help users estimate their physical activity based on the International Physical Activity Questionnaire (IPAQ). Functioning issues are quantified using the Numeric Rating Scale (NRS) for each category in the brief ICF core set for osteoarthritis, following a biopsychosocial approach.

An innovative feature of the “Move for Knee” ™ app compared to the similar digital technologies (18–21) is the self-assessment of physical activity levels using MET (Metabolic Equivalent of Task). Users can complete the questionnaire multiple times throughout their treatment, with reminders sent every two weeks to reevaluate their activity levels. The app also delivers a personalized experience by providing detailed exercise descriptions and a motion capture feature that enables patients to record and share videos of their exercises with healthcare providers, ensuring proper execution.

Another significant advantage of the app is its chat function, addressing a common limitation in other applications (18–21). This feature offers direct communication with healthcare professionals providing valuable support for patients facing barriers, such as limited facility access or transportation difficulties. By promoting a healthier lifestyle, the app may reduce flare-ups, decrease medication use, lower work absences, and increase productivity. Improved management of pain and joint function can further reduce the need for costly interventions, such as surgery, intensive physiotherapy, or long-term medication, potentially benefitting both direct and undirect healthcare costs.

Finally, when comparing the development processes of the Move Your Knee app with other existing apps, such as the Knee Osteo-Arthritis Lifestyle App (iKOALA), it is evident that the KOALA App was developed in two phases: planning (which included literature review and qualitative data collection) and optimization (involving user trials and feedback) (22).

The KOALA app emphasizes a person-based approach (PBA) to development, ensuring that user needs and barriers are central to the design process. Moreover, it provides comprehensive lifestyle management, including guidance on physical activity, education, and social support.

In contrast, the Move Your Knee app focuses on delivering personalized exercise programs based on self-reported data, integrating real-time tracking and feedback mechanisms. It also incorporates self-assessment tools and exercise programs grounded in MET, featuring a motion capture function that allows users to record their exercises, thereby facilitating remote monitoring by healthcare providers.

Another significant difference lies in the study design and outcome measures. The KOALA app emphasizes iterative development and continuous user engagement throughout the process, focusing on qualitative user experience and usability evaluation during the development phase. It plans to conduct randomized controlled trials to validate the app in real-world settings after making revisions based on user feedback.

Conversely, the Move Your Knee app aims to conduct a double-arm interventional trial that compares the app's effectiveness against a control group receiving standard advice for physical activity. It structures a precise and concise protocol according to quantitative outcome measures assessed at multiple time points (30, 90, and 180 days) to evaluate pain, stiffness, function, and quality of life. Additionally, it employs a structured feedback mechanism for the intervention group to gather insights on user experience and any adverse effects. The Move Your Knee app also sets defined inclusion criteria, targeting men and women aged 45–70 years with unilateral or bilateral tibiofemoral KOA diagnosed according to established criteria.

In summary, while the iKOALA app strongly emphasizes user-centered design and iterative feedback for development, the Move Your Knee app adopts a more structured clinical trial approach to evaluate its effectiveness against a control group. The choice of methodologies and outcome measures reflects their respective research objectives, with one focusing on development and usability, and the other on clinical effectiveness and patient outcomes.

However, there are potential limitations to the clinical application of this app. These include reduced usability among very elderly or cognitively impaired individuals and low motivation for sustained app use. Previous studies have reported dropout rates as high as 20% (11), particularly when users fail to perceive tangible benefits or lack sufficient support. Our proposal aims to address these challenges by incorporating motivational support strategies. The reliance on self-reported data is another limitation, as it allows for immediate tracking but may be subject to biases, leading to inaccuracies in physical activity reporting and potentially affecting the evaluation of intervention outcomes. Future iterations of the app could include educational strategies, particularly focusing on nutritional advice.

Considering that the primary goals of therapeutic interventions for KOA are pain relief, improved joint function, and enhanced quality of life (23), the next steps in the app's development will involve a RCT. This study will assess the efficacy of the intervention compared to educational advice by measuring changes in the BPI, WOMAC, and EQ-5D among KOA patients.

## 5 Conclusions

The “Move for Knee”™ app represents a promising, accessible, and cost-effective component of the multimodal approach for managing KOA. By integrating personalized care, real-time monitoring, and improved communication between patients and healthcare providers, the app has the potential to significantly enhance the overall management of the condition.

Future research should prioritize not only evaluating the app's clinical efficacy but also ensuring its accessibility and usability to maximize its impact across different KOA phenotypes.
